# Differential effects of childhood maltreatment types and timing on psychopathology in formerly out-of-home placed young adults

**DOI:** 10.1192/j.eurpsy.2025.10127

**Published:** 2025-10-23

**Authors:** Maria Meier, Inga Schalinski, Cyril Boonmann, Nils Jenkel, Süheyla Seker, Delfine d’Huart, Jörg M. Fegert, Vera Clemens, Marc Schmid, David Bürgin

**Affiliations:** 1 Child and Adolescent Psychiatric Research Department, University Psychiatric Clinics Basel (UPK), University of Basel, Basel, Switzerland; 2 Department of Psychology, University of Konstanz, Konstanz, Germany; 3 Faculty of Human Sciences, Institute of Psychology, Universität der Bundeswehr München, Neubiberg, Germany; 4 Department of Child and Adolescent Psychiatry (LUMC Curium), Leiden University Medical Center, Leiden, The Netherlands; 5 Department of Forensic Child and Adolescent Psychiatry, University Psychiatric Clinics Basel (UPK), University of Basel, Basel, Switzerland; 6 Department of Social Work, Stockholm University, Stockholm, Sweden; 7 Department of Child and Adolescent Psychiatry/Psychotherapy, University of Ulm, Ulm, Germany; 8 Jacobs Center for Productive Youth Development, University of Zurich, Zurich, Switzerland

**Keywords:** childhood maltreatment, conditional random forest regression, HiTOP, machine learning, psychopathology

## Abstract

**Background:**

Childhood maltreatment (CM) increases the risk for psychopathology and CM type, severity and timing are considered important modulating factors in this relationship. However, reported associations are heterogeneous and hardly considered vulnerable groups broadly exposed to CM.

**Methods:**

We investigated the association between CM types and timing and psychopathology in formerly out-of-home placed young adults (*N* = 185; 32% women, age mean = 26.38 years, *SD* = 3.49). CM was assessed using the Maltreatment and Abuse Chronology of Exposure Scale. Conditional random forest regression was used to estimate the importance of CM types (abuse, neglect, peer victimization, and sexual abuse), timing (ages 3–18), and global measures (severity, multiplicity, and duration) on adult general, internalizing, and externalizing problems (Achenbach System of Empirically Based Assessment). We validated the results using diagnoses of mental disorders clustered with the Hierarchical Taxonomy of Psychopathology model.

**Results:**

Global CM measures were stronger predictors of internalizing problems than CM type and timing. Abuse in early childhood was a stronger predictor of externalizing problems compared to global CM measures.

**Conclusions:**

Considering CM type and timing might be valuable to guide maltreatment-informed interventions in therapeutic settings.

## Introduction

Childhood maltreatment (CM), including interpersonal victimization, abuse, and neglect, is a major public health concern and a strong risk factor for mental and physical diseases [1–4]. CM is highly prevalent and associated with severe health issues and financial costs [5–7], making it a key target for prevention and intervention efforts. Individuals exposed to CM are at increased risk of a broad range of mental disorders, especially internalizing (e.g., mood and anxiety disorders or stress-related disorders) and externalizing disorders (e.g., substance use or disruptive behavior disorder). [6, 8–11], likely mediated through transdiagnostic mechanisms [3, 12]. They tend to exhibit earlier symptom onset, higher symptom severity and comorbidity, increased suicidal ideation, and poorer treatment response compared to individuals with the same diagnosis but without CM exposure [13–16].

Research on the sequelae of CM often focused on global CM scores that aggregate the severity of different CM types (e.g., sexual abuse) [17–19]. This approach revealed dose–response and type-specific effects [20–22]. However, the frequent co-occurrence of exposure types constrains the inferences. Moreover, global CM scores conceal temporal information, limiting the exploration of questions such as whether CM has a stronger impact on health during sensitive developmental periods [23, 24].

For this reason, analyses need to acknowledge the type, timing, and duration of CM exposure. While commonly used CM measures rarely capture timing [17, 25], some newer measures do (e.g., the Maltreatment and Abuse Chronology of Exposure scale [MACE], the International Society for the Prevention of Child Abuse and Neglect [ISPCAN] Child Abuse Screening Tool [ICAST-R], [26] and the Stress and Adversity Inventory [STRAIN]) [27–29]. Research using these measures revealed that earlier exposure is often predictive of later exposure [30, 31]. Making use of them may further our understanding of developmental risks, and help to target prevention and intervention efforts.

Nevertheless, analyses combining CM types and timing are hitherto scarce [32], likely because the complex dependencies of the data commonly violate the assumptions of widely used statistical tests. To remedy this, recent reports have utilized machine learning algorithms, such as conditional random forest regressions [33–36]. While these analyses were based on data from patient populations, e.g., transdiagnostic inpatients [33] or individuals suffering from PTSD [34], no study has investigated the differential impact of types and timing of CM on psychopathology in individuals formerly placed in youth residential out-of-home care. This highly vulnerable group is underrepresented in research despite high rates of CM exposure [37, 38].

We thus aimed to investigate how type, timing, duration, multiplicity, and severity of CM predict general, internalizing, and externalizing psychopathology in a sample of young adults formerly placed in youth residential care [6, 8–11]. We used conditioned random forest regression to consider all predictors simultaneously and test which of the CM characteristics were most important to predict psychopathology in young adulthood. Furthermore, we used competitive hypothesis testing and contrasted whether the importance of type and timing exceeded the importance of global indicators of CM.

## Methods

### Sample and procedure

The sample stemmed from the Swiss Study for Clarification and Goal-Attainment in Child Welfare and Juvenile Justice Institutions (German: Modellversuch Abklärung und Zielerreichung in stationären Massnahmen, MAZ.) assessed from 2007 to 2012. It comprised *N* = 592 participants aged 5 to 27 (*mean* age = 15.86 years, *SD* = 2.99 years; 32.1% female) from 64 residential care institutions in Switzerland [39]. Between 2018 and 2020, the follow-up study Youth Welfare Trajectories: Learning from Experience (German: Jugendhilfeverläufe: Aus Erfahrung Lernen, JAEL) was conducted on a subset of participants, who completed questionnaires via an online platform (www.weaskyou.ch) and were invited to (semi-structured clinical) interviews in person [40]. The cross-sectional analyses presented here are based on data of *n* = 185 participants (mean age = 26.38 years, *SD* = 3.49 years, range = 16–38 years; 34% women) who took part in the face-to-face assessments. This sample size is comparable to previous studies in clinical and high-risk samples that used conditional random forest regression (reported sample sizes ranging from 43 to 341, with approximately half of them reporting samples of up to 200 participants) [33–36, 41–47]. Included participants neither differed from non-included participants in psychosocial characteristics and mental health burden assessed at baseline (MAZ., see Supplemental Table S1) nor in regard to follow-up data of criminal records from the Swiss Federal Bureau of Statistics (BFS) up until the year 2017 (see Supplemental Table S2).

Participants provided written informed consent and received vouchers of up to CHF500. The study was conducted in accordance with the Declaration of Helsinki and approved by the Ethics Commission of Northwestern Switzerland (EKNZ, Ref. 2017–00718). Findings on the links between CM and personality functioning [48], neurobiological markers [49–53], and the emergence and stability of psychopathology [13] are published elsewhere.

### Measures

#### Childhood maltreatment

We used the Maltreatment and Abuse Chronology of Exposure (MACE-X) scale to assess the severity and timing of CM [28]. For each of the 75 items, participants reported whether and at what age (from 1 to 18) they experienced it (e.g., parents or caregivers living in the household did hurtful things like insulting you). Based on this, exposure to 10 forms of CM was assessed, which were summarized to four CM types to ease interpretability: parental abuse (ABUSE, summarizing parental physical and verbal abuse, parental non-verbal emotional abuse, witnessed physical violence toward parents, and witnessing violence toward siblings), parental neglect (NEGLECT summarizing emotional and physical neglect), violence by peers (PEER summarizing peer emotional and peer physical violence), and sexual abuse (SEXA, indexed by the subscale familial and non-familial sexual abuse). The severity of CM type per life year was indexed as *weighted* score (ranging from zero to 10) to ensure comparability across the four CM types. Additionally, we computed three global measures of CM. For doing so, we used previously suggested thresholds [54, 55] from validation studies. The three global measures comprised: duration (DURATION, range: 0–18 years), indicating how many years participants were exposed to at least one of the ten original CM types above threshold [54, 55]; severity across all ten types of CM (SEVERITY, range: 0–100); and multiplicity (MULTI, range: 0–10), indicating the number of ten CM types experienced above threshold [54, 55]. CM types (ABUSE, NEGLECT, PEER, SEXA) per year (3–18) and global measures (DURATION, SEVERITY, MULTI) were used as predictors in the conditional random forest regression. Similar to previous studies, [34, 35] we excluded reports of years one and two due to their low reliability (infantile amnesia). [28, 34] Including these years did not change the interpretation of results.

#### Mental health problems

Participants filled in the Achenbach System of Empirically Based Assessment (ASEBA; the Youth Self-Report [YSR; < 18 years; *N* = 5] and the Young Adult Self-Report [YASR; > 18 years; *N* = 180]) [56, 57], consisting of 118 or 124 items respectively, which were answered on a 3-point Likert scale (0 = not true, 1 = somewhat or sometimes true, 2 = very or often true). We used the total score, indicating *overall problems*, and the subscales *internalizing* and *externalizing problems.* Raw values were transformed to *T*-values [56, 57], with values above 60 indicating clinically relevant symptomatology. *T*-values were used as outcomes in the conditional random forest regression.

#### Diagnoses of mental disorders

To complement the self-report data, we further employed the Structured Clinical Interview for the Diagnostic and Statistical Manual (DSM) of Mental Disorders 5th revision DSM-5 Disorders – Clinician Version (SCID-5-CV). The SCID-5-CV is a semi-structured clinical interview assessing the adult disorder dimensions outlined in the DSM-5. Responses were recorded (1 = presence, 0 = absence), and identified diagnoses were categorized according to the F-codes of the *International Statistical Classification of Diseases and Related Health Problems 10th edition* (ICD-10). Following the Hierarchical Taxonomy of Psychopathology (HiTOP) model, we scored whether the criteria for a current mental disorder were fulfilled, and grouped ICD-10 codes into *internalizing* (ICD-10 F32–F34, F38–F38, F41, F43, F40–F42, F50.1, F50.3, F51, F60.31, F93.3, F93.8, F93) and *externalizing* diagnoses (F10–F25, F60.2, F60.3, F60.4, F60.81, F60.0, F63.2, F90–F92, F94). Unlike traditional diagnostic systems, HiTOP focuses on psychopathological syndromes and broader spectra based on the observed co-occurrence of symptoms. For the analysis, we used the variables *any diagnosis* (1 = presence, 0 = absence), at least one *internalizing disorder* (1 = presence, 0 = absence), at least one and *externalizing disorder* (1 = presence, 0 = absence).

### Statistical analysis

Analyses were performed in R version 4.3.0 and RStudio version 2023.3.0.386 using the packages *haven* [58], *lm.beta* [59], *party* [60–62], and *caret* [63]. Figures were created using *ggplot2* [64], *corrplot* [65], and *patchwork* [66]. Pearson’s correlation coefficients were calculated for descriptive purposes. Three conditional random forest regressions were modeled to predict *overall*, *internalizing*, and *externalizing problems* using the global (DURATION, SEVERITY, MULTI) and type- and timing-specific CM measures (ABUSE, NEGLECT, PEER, SEXA per life year between three and 18) while controlling for sex and age. We competitively tested the importance of global CM measures against timing- and type-specific measures. In linear regression models, a normal distribution of residuals is assumed, and a high collinearity critically limits the interpretability of findings; conditional random forest regressions do not rely on specific distributions and can handle a high number of collinear predictors [61, 62]. Hence, they have repeatedly been used to analyze the type- and timing-specific effects of CM on psychopathology and other outcomes [33, 36]. Random forest regressions are a machine learning approach that estimates a specified number of decision trees (“forest”) by randomly drawing a subset of the data and a subset of the predictor variables for consideration at the nodes. To avoid overfitting, the model is trained on 75% of the data and evaluated on the remaining 25%. We modeled the conditional random forest regression as described earlier [33] and estimated each predictor’s *variable importance* (VI) by sequentially permuting each predictor in the model, refitting the random forest, and determining the extent to which each permutation increased the mean square error (MSE), an index of the goodness of fit of the model. While permuting important predictors results in larger increases in MSE, permuting unimportant predictors should have a negligible effect. We used a total of 100 permutations with randomly drawn training and test data and subsequently averaged the VI estimates. To obtain the statistical significance of each VI, the random forest analysis was run 5,000 times using reshuffled outcome measures to calculate random chance VIs and a *SD* for each predictor. We used *Z*-tests to derive the probability of observing a high VI by chance.

To validate the results, we used the CM measures that were significant predictors in the conditional random forest regression and tested whether the strongest predictor per CM type could predict mental disorder status in adulthood using binomial generalized linear models.

## Results

### Descriptive analyses

**Figure 1.**
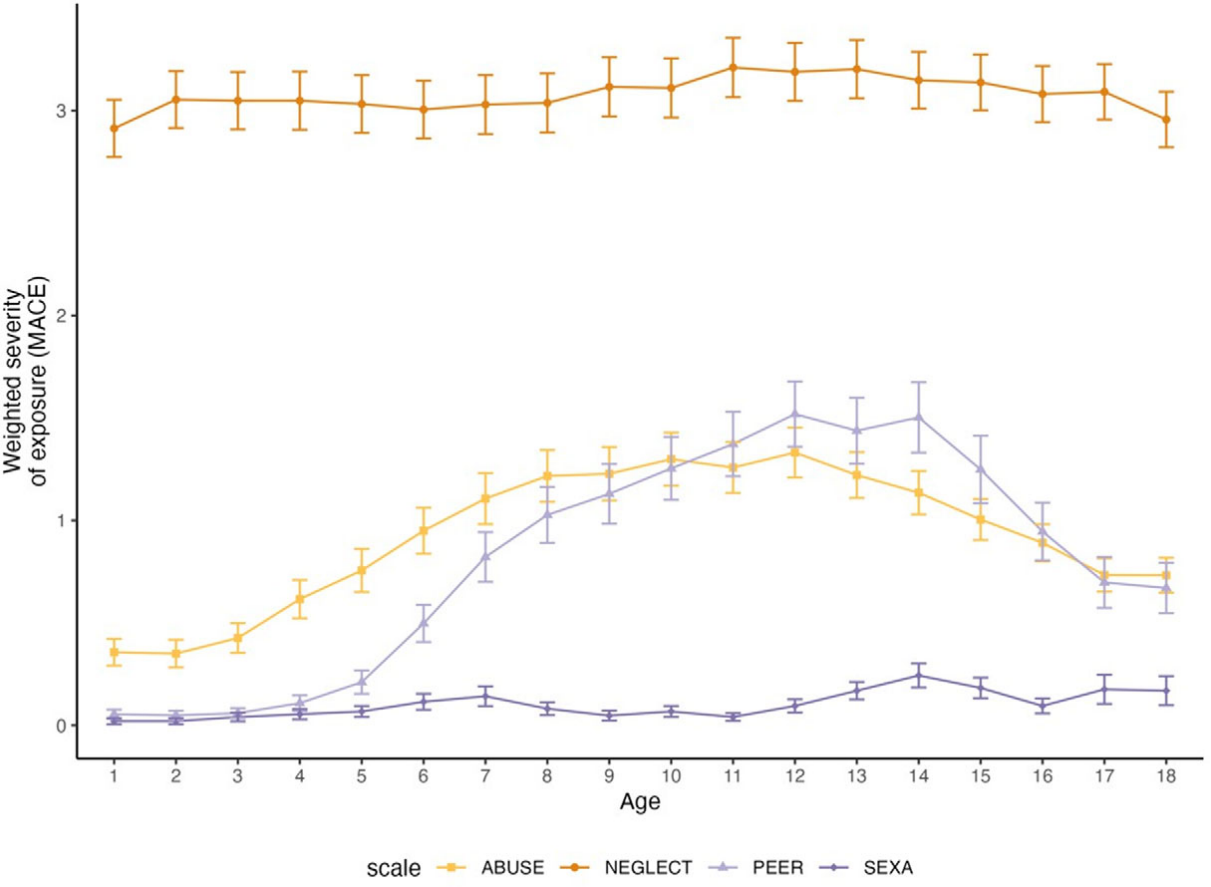
Weighted severity of exposure scores (theoretical range 0–10) per life year and category of childhood maltreatment, i.e., parental abuse (ABUSE) and neglect (NEGLECT), peer violence (PEER), and sexual abuse (SEXA). Error bars represent *SE.*

Sample characteristics are listed in Table 1. From *N* = 185 participants, 87.6% experienced at least one CM type (ABUSE, NEGLECT, PEER, and SEXA) above-threshold [54, 55]. Mean severity of each type per year can be derived from Figure 1 and prevalence rates can be derived from Supplemental Table S3. The mean prevalence of peer violence was less than 10% for ages three to five, and the mean prevalence of sexual abuse was less than 10% for ages three to 18, which may comprise the informativeness of the models in this regard.

**Table 1.** Description of the sample

Variable	Sample (*n* = 185)
Age (in years) in M (SD)	26.38 (3.49)
Sex (self-reported) in % [female/male]	34/66
Country of Birth in %	
Switzerland	79.46
Other European country	8.11
Latin America	5.95
Africa	3.24
Other	1.62
Age at first placement in years in M (SD)*	11.55 (4.9)
Age at leaving the last institution in M (SD)*	18.77 (3.05)
Duration between first placement and last placement termination in years in M (SD)*	7.06 (5.09)
Reason for out-of-home placement at initial assessment in % [civil/criminal/other]	47.03/25.41/23.78
Number of placements in M (SD)*	3.85 (3.95)
Self-reported number of premature terminations of placements in M (SD)*	0.77 (1.83)
Number of individuals ever placed in a foster family in % [yes/no]*	37.84/62.16
Childhood maltreatment (MACE)	
Duration in years (DURATION) in M (SD)	10.54 (7.36)
Multiplicity (MULTI) in M (SD)	3.03 (2.42)
Severity (SEVERITY) in M (SD)	30.72 (17.34)
Average prevalence of parental neglect (NEGLECT) in %	91
Average prevalence of parental abuse (ABUSE) in %	54
Average prevalence of peer violence (PEER) in %	27
Average prevalence of sexual abuse (SEXA) in %	5
Mental health problems (ASEBA)	
Overall problems (T score > 60) in %	31
Internalizing problems (T score > 60) in %	31
Externalizing problems (T score > 60) in %	25
Mental disorders (HiTOP)	
Internalizing diagnosis in %	32
Externalizing diagnosis in %	54

*Note*: *These placement-related characteristics were self-reported retrospectively in young adulthood as part of the JAEL study.

Participants with any diagnosis reported significantly more overall mental health problems (ASEBA total *mean* = 57.46, *SD* = 10.31) compared to participants without a diagnosis (*mean* = 48.97, *SD* = 9.45), *t*(160.94) = −5.73, *p* < .001, *d* = −0.85. Participants suffering from an internalizing disorder reported significantly more internalizing problems (ASEBA internalizing *mean* = 60.13, *SD* = 10.23) compared with those without an internalizing diagnosis (*mean* = 49.63, *SD* = 10.03), *t*(114.77) = −6.57, *p* < .001, *d* = −1.04, and participants suffering from an externalizing disorder reported more externalizing problems (ASEBA externalizing *mean* = 56.17, *SD* = 10.68) compared with their counterparts (*mean* = 50.00, *SD* = 9.02), *t*(181.96) = −4.25, *p* < .001, *d* = −0.62.

Overall, 61% fulfilled the criteria for at least one mental disorder, with 25.95% being diagnosed with both an internalizing and externalizing disorder, 6.49% with an internalizing, and 27.57% with an externalizing disorder, and 39.46% with neither. Global and type- and timing-specific CM measures were significantly interrelated and associated with self-reported mental health problems (cf. correlation matrix in Supplemental Figure S1).

### Predicting overall mental health problems from CM type and timing

**Figure 2.**
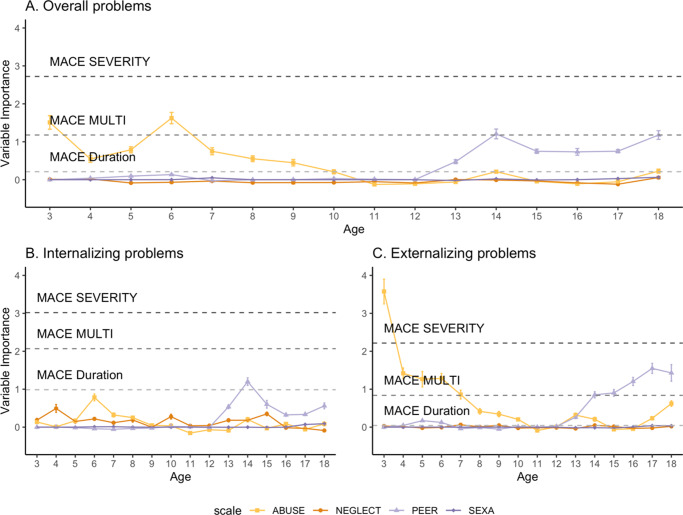
Variable importance of the timing-specific effects of different childhood maltreatment types (solid lines) and the global childhood maltreatment severity, multiplicity, and duration measures (dashed lines) for overall mental health problems (A), internalizing mental health problems (B), and externalizing mental health problems (C). High variable importance implies that the variable is a strong predictor of the respective subscale of self-reported mental health problems. MACE SEVERITY = severity of exposure. MACE MULTI = number of individual scales above cut-off. MACE Duration = duration of above-threshold exposure.

Parental abuse during ages three, five, six, and seven (early to middle childhood) significantly predicted overall mental health problems, with the strongest predictor being parental abuse at age six (VI = 1.63, *p* = .002). During adolescence, sexual abuse at age 18 (VI = 0.06, *p* = .020), and peer violence throughout ages 14 to 18 significantly predicted overall mental health problems (strongest predictor: peer violence at age 14, VI = 1.21, *p* = .009). Global CM severity (VI = 2.72, *p* < .001) and multiplicity (VI = 1.18, *p* = .009) significantly predicted overall mental health problems. Parental neglect and CM duration were no significant predictors. Estimates of the VI of all predictors are depicted in Figure 2A and in Supplemental Table S4.

Parental abuse at age six significantly predicted disorder status (HiTOP any disorder; OR = 1.30, *beta* = 0.69, *SE* = .13, *p* = .034) when controlling for sex and age. Neither sexual abuse at age 18 (OR = 1.00, *beta* = 0.01, *p =* .988), peer violence at age 14 (OR = 1.79, *beta* = 0.58, *SE* = 0.074, *p =* .068), severity (*OR* = 1.74, *beta* = 0.55, *SE* = 0.01, *p* = .097), nor multiplicity (OR = 1.65, *beta* = 0.50, *SE* = 0.07, *p* = .133) predicted disorder status.

### Predicting internalizing problems from CM type and timing

Parental abuse at age six (VI = 0.78, *p* = .027) and sexual abuse at ages 17 and 18 significantly predicted internalizing problems, with severity at age 18 being the stronger predictor (VI = 0.09, *p* = .008). Peer violence during adolescence, at ages 14, 15, and 18 significantly predicted internalizing problems (strongest predictor was PEER at age 14, VI = 1.2, *p* = .007). CM severity (VI = 3.02, *p* < .001), multiplicity (VI = 2.07, *p* < .001), and duration (VI = 1.78, *p* = .006) as well as sex (VI = 1.78, *p* = .006) significantly predicted internalizing problems. Parental neglect was no significant predictor. Global CM measures were stronger predictors of internalizing problems as compared with the strongest type- and timing-specific effect (PEER at age 14). Estimates of the VI of all predictors are depicted in Figure 2B and Supplemental Table S5.

Parental abuse at age six (OR = 3.31, *beta* = 1.20, *SE* = .12, *p* < .001) and peer violence at age 14 (OR = 3.49, *beta* = 1.25, *SE* = .07, *p* < .001) significantly predicted internalizing disorder status (HiTOP internalizing disorder) when controlling for sex and age. Sexual abuse at age 18 was no significant predictor of internalizing disorder status (*p* > .05), but all global measures were significant predictors (SEVERITY: OR = 3.31, *beta* = 1.20, *SE* = .01, *p* < .001; MULTI: OR = 2.70, *beta* = 0.99, *SE* = .07, *p* = .006; DURATION: OR = 2.84, *beta* = 1.04, *SE* = .02, *p* = .003).

### Predicting externalizing problems from CM type and timing

Externalizing problems were significantly predicted by parental abuse during early to middle childhood, i.e., at ages three, four, five, six, and seven, with the strongest predictor being parental abuse at age three (VI = 3.57, *p* < .001). In addition, sexual abuse at age 18 significantly predicted externalizing problems (VI = 0.03, *p* = .043). Moreover, experiencing peer violence at ages 14–18 (i.e., during adolescence) significantly predicted externalizing problems, with the strongest predictor being peer violence at age 17 (VI = 1.55, *p* = .001). CM severity (VI = 2.22, *p* = .001), and multiplicity (VI = 0.84, *p* = .025) significantly predicted externalizing problems. Parental neglect and CM duration did not significantly predict externalizing problems. The strongest type- and timing-specific effect (ABUSE at age three) was a stronger predictor of externalizing problems as compared with the global measures. Estimates of the VI of all predictors are depicted in Figure 2C and listed in Supplemental Table S6.

Parental abuse at age three (OR = 2.05, *beta* = 0.71, *SE* = .22, *p* = .025) and peer violence at age 17 (OR = 1.90, *beta* = 0.64, *SE* = .11, *p* = .041) were significant predictors of externalizing disorder status (HiTOP externalizing disorder) when controlling for sex and age. However, sexual abuse at age 18 and global CM severity, multiplicity, and duration did not significantly predict externalizing disorder status (all *p* > .05).

## Discussion

We investigated the impact of types and timing of CM on general, internalizing, and externalizing psychopathology among young adults with previous experience of residential out-of-home care. Using conditional random forest regressions, we found that CM severity and multiplicity were important predictors of general psychopathology, next to type- and timing-specific effects of peer abuse in mid-to-late adolescence and sexual abuse in late adolescence. CM severity and multiplicity were the most important predictors of internalizing problems, followed by peer victimization in mid-to-late adolescence, with minor effects of early parental and late adolescent sexual abuse. Regarding externalizing problems, CM severity, parental abuse in early childhood, and effects of peer violence in mid-to-late adolescence were most important, with minor effects of sexual abuse in late adolescence. Most effects were replicated using data from clinical interviews. Our findings add a nuanced perspective on the association between CM and psychopathology in young adults with a history of youth residential care in Switzerland.

The high severity and co-occurrence of reported CM (87.6%) align with those of other samples of young adults in and after out-of-home care [37, 67–69]. Studies using the same CM measure found comparable rates in psychiatric inpatients (90%) [33], slightly higher rates in Norwegian adults with previous youth residential care placements (98%) [70], and substantially lower rates in the healthy community (51.4%) [36]. Rates of mental disorders (61%) were higher compared to those of the general population (18%) and slightly higher compared to adults previously placed in out-of-home care (31–45% depending on the type of out-of-home care) [71]. In our study, 32% fulfilled the criteria for an internalizing diagnosis and 54% for an externalizing diagnosis, with 26% fulfilling the criteria for both. This lines up with results indicating that externalizing disorders are more common than internalizing disorders in out-of-home-placed individuals, particularly when placed within juvenile justice institutions [71]. Our findings underline the need for intensified prevention efforts, suitable psychosocial and therapeutic support, and targeted interventions in this vulnerable population.

Confirming prior evidence of a dose–response relationship between CM and psychopathology [6, 8–11], global CM measures were strong predictors of mental health problems, with larger effects for internalizing than externalizing problems, likely due to its impact on emotional functioning (e.g., shame, guilt, insecurity) [72, 73], self-concept (e.g., negative self-perception, impaired self-esteem) [74, 75], and the neurobiological systems involved in emotion and stress regulation (e.g., limbic system) [76, 77]. Similar results have also been reported for other youth residential care cohorts [37, 68, 78].

Beyond the global effects of CM, we found timing-specific effects, particularly regarding abuse in early childhood and peer victimization in late adolescence, with stronger effects on externalizing than internalizing symptoms. While considerable variability in reported sensitive periods for internalizing and externalizing disorders exists, there is little consistency in the periods identified for specific CM types or categories (e.g., abuse vs. neglect) [32]. For depression, cumulative effects of CM seem to be most pronounced in mid-childhood [79]. In contrast, we found that type- and timing-specific effects were strongest in early childhood and mid-to-late adolescence, which might be related to our sample of youths in residential care, who were oftentimes placed to prevent maltreatment within the family context. As such, residential care placements might exert their most protective effects against CM in mid-childhood and early adolescence but might be prone to further adverse peer victimization.

Of note, sexual abuse was rarely reported in our sample, critically limiting the conclusions that could be drawn on this CM type. Additionally, experiences of neglect were reported by approximately 90% of participants throughout ages 3 to 18 (see Supplementary Table S3), contributing substantially to the global CM measures and limiting the probability of detecting type- and timing-specific effects in this regard due to possible ceiling effects. As such, our sample is characterized by high prevalence rates of parental neglect throughout childhood and adolescence and overall low prevalence rates of sexual abuse. Such ceiling and floor effects may substantially impact the results of random forest regressions, as they artificially restrict the variance of a predictor, diminishing the informative value for meaningful sample splits, and thereby distorting the estimation of variable importance [62]. It must therefore be stressed that the finding of parental neglect *not* being a significant predictor of mental health outcomes is likely due to the given exposure distribution in our sample and the associated bias introduced to the random forest regression model.

While not all findings concerning the externalizing dimension could be replicated, this might be related to the poorer overlap between self-reported externalizing problems assessed by the ASBEA and the clinically assessed externalizing diagnoses grouped by the HiTOP model. While the latter subsumes substance use disorders (a common diagnosis in our sample), the ASEBA’s externalizing domain only contains a few items on substance misuse. In sum, while the literature on timing effects is steadily growing, sensitive periods for psychiatric disorders and related endpoints do not converge on single periods of vulnerability [32]. Our work adds novel type- and timing-specific insights in a highly vulnerable subgroup of young adults with previous youth residential care placements.

Our results showcase the complex CM trajectories of institutionalized individuals and the significance of diverse maltreatment experiences that may derive from parents in early childhood (i.e., before placement) as well as from peers in adolescence (i.e., during placement). Even though individuals in our sample were first placed in an institution at an average age of 11 and stayed in out-of-home placements for an average of 7 years thereafter, the majority of the sample was still in contact with their families of origin throughout their stays. This may explain the high chronicity of CM exposure despite the intervention of youth welfare institutions. This moreover, underlines the need to routinely and systematically screen for CM in youth residential care. Doing so might help to integrate safeguarding measures [79], timely enroll individuals in therapeutic and pedagogical interventions, and foster feelings of safety and connectedness [80, 81]. Our results suggest that prevention and intervention strategies might specifically tackle the potential intergenerational transmission of externalizing behaviors, such as aggression and violence within families [16, 82, 83], and cycles of violence and revictimization in peer groups. This is a complex endeavor, as strategies may need to target various contexts and groups of perpetrators simultaneously. By doing so, one needs to critically appraise the potential victim–offender overlap [84], but targeted, personalized approaches might prove helpful in breaking vicious interdependencies [85, 86]. It should thereby not be overlooked that neglect was the predominant CM type experienced in this population and targeting neglect, starting in early childhood, may therefore have a strong and lasting reducing impact on overall CM severity.

Beyond screening and trauma-informed interventions within the care system, it is important to prevent CM from happening in the first place and to implement universal, indicated, and selective prevention programs. Universal programs such as Triple P (Positive Parenting Program) and family-centered approaches like (Early) Head Start have been shown to significantly reduce aversive parenting practices and the risk of abuse at the population level [87, 88]. For example, Triple P consistently reduced harsh parenting and improved positive parenting [87], and Head Start reduced rates of abuse in young children [88]. In cases in which CM is already evident, Multisystemic Therapy for Child Abuse and Neglect (MST-CAN) serves as a robust intervention, with strong evidence showing that it reduces abusive and neglectful parenting, parental distress, and the need for out-of-home placements [89, 90]. Together, these evidence-based approaches demonstrate that for the effective reduction of CM, a combination of early universal prevention, indicated, selective prevention, as well as targeted intervention is urgently needed.

There are some limitations to our study. First, the retrospective self-report CM measure may be subject to biases [91–93]. Indeed, there is poor overlap between prospective versus retrospective CM measures [94]. While subjective reports might weigh more heavily in relation to psychopathology [19, 93], the validity might be limited in our sample. Importantly, while the MACE-X separates different perpetrators (e.g., peers, siblings, parents), “parent” is introduced as “an adult with whom one lived at that time.” Thus, it was unclear whom the participants referred to specifically and forms of abuse experienced in the family of origin (e.g., on weekend leaves) and beyond (e.g., inside or outside the institution) might be mixed. Second, the sample was heterogeneous regarding individual residential care paths and received interventions. Due to restrictions in sample size, we refrained from addressing this heterogeneity (e.g., by analyzing subsamples). While the sample is fairly representative of out-of-home care institutions in Switzerland [95], and there appears to be no systematic bias between included and non-included participants (see Supplemental Tables S1 and S2), we cannot rule out the possibility that participants in non-included institutions, all those who did not participate at baseline, or participants who dropped out after baseline were more burdened than included participants, which might bias our results.

Despite these shortcomings, our study has several strengths: We investigated a hard-to-reach and vulnerable group of young adults with a history of youth residential care using a comprehensive measure of CM and transdiagnostic measures of mental health problems and psychopathology. We employed a state-of-the-art machine learning algorithm to handle a high number of multicollinear predictors indexing CM type and timing on general, internalizing, and externalizing problems. We validated our findings using clinical diagnoses and applied competitive hypothesis testing to contrast the importance of global CM measures to type- and timing-specific predictors [32].

Our findings may convey important implications for universal prevention, targeted interventions and decision-making in health care systems and child welfare policies. Complementing the prevention of transdiagnostic risk factors, such as CM, the promotion of accessible and suitable interventions for vulnerable populations in various care settings might counteract some maltreatment-associated risks [96], especially if type- and timing-specific CM reports are acknowledged. Preventing CM and reducing its overall severity, especially regarding early parental and domestic abuse and later peer revictimization, might be an important target to improve mental health among out-of-home placed children and adolescents.

## Supporting information

10.1192/j.eurpsy.2025.10127.sm001 Meier et al. supplementary material Meier et al. supplementary material

## Data Availability

Data are available upon request.

## Supplementary material

The supplementary material for this article can be found at http://doi.org/10.1192/j.eurpsy.2025.10127.
