# Supplementary material for: Differential effects of childhood maltreatment types and timing on psychopathology in formerly out-of-home placed young adults
Source: Eur Psychiatry. 2025 Oct 23;68(1):e160. doi: 10.1192/j.eurpsy.2025.10127 (PMC12646124; doi:10.1192/j.eurpsy.2025.10127)
Supplement: Meier et al. supplementary material [file S0924933825101272sup001.docx]

***Supplements for* Differential effects of type and timing of childhood maltreatment on psychopathology in formerly out-of-home placed young adults**

Maria Meier ^1, 2^, Inga Schalinski ^3^, Cyril Boonmann ^1, 4, 5^, Nils Jenkel ^1^, Süheyla Seker ^6^, Delfine d’Huart ^1, 5^, Jörg M. Fegert ^7^, Vera Clemens ^8^, Marc Schmid ^1^, David Bürgin ^1, 9^

^1^ Child and Adolescent Psychiatric Research Department, University Psychiatric Clinics Basel (UPK), University of Basel, Switzerland
^2^ Department of Psychology, University of Konstanz, Konstanz, Germany
^3^ Universität der Bundeswehr München, Faculty of Human Sciences, Institute of Psychology, Neubiberg, Germany
^4^ Department of Child and Adolescent Psychiatry (LUMC Curium), Leiden University Medical Center, The Netherlands
^5^ Department of Forensic Child and Adolescent Psychiatry, University Psychiatric Clinics Basel (UPK), University of Basel, Switzerland

^6^ Department of Social Work, Stockholm University, Stockholm, Sweden

^7^ Department of Child and Adolescent Psychiatry/Psychotherapy, University of Ulm, Germany
^8^ Jacobs Center for Productive Youth Development, University of Zurich, Zurich, Switzerland

Correspondence: [maria.meier@unibas.ch](mailto:maria.meier@upk.ch)

Table of Contents

[Table S1. 3](#_Toc207815208)

[Table S2. 4](#_Toc207815209)

[Table S3. 5](#_Toc207815210)

[Figure S1. 6](#_Toc207815211)

[Table S4. 7](#_Toc207815212)

[Table S5. 9](#_Toc207815213)

[Table S6. 12](#_Toc207815214)

Table S1. Comparison of psychosocial characteristics and mental health burden between included (*N* = 185) versus non-included participants (*N* = 412) at baseline (MAZ.).

|  | *Included* | *Non-included* | *Test-statistic* | *p-value* |
| --- | --- | --- | --- | --- |
|  | M (SD) or N (%) | M (SD) or N (%) |  |  |
| Age | 16.47 (2.64) | 16.16 (2.55) | *t*(293.3) = -1.24 | 0.217 |
| Sex (Female) | 62 (33.5%) | 132 (32%) | χ^2^=0.068, df=1 | 0.794 |
| Nationality (Swiss) | 160 (88.9%) | 341 (82.8%) | χ^2^=3.154, df=1 | 0.076 |
| Reason for placement (t1) |  |  | χ^2^=3.067, df=2 | 0.216 |
| Civil | 87 (48.9%) | 225 (55.8%) |  |  |
| Criminal | 47 (26.4%) | 101 (25.1%) |  |  |
| Other | 44 (20.8%) | 77 (19.1%) |  |  |
| Any personality disorder^1^ | 35 (25.4%) | 73 (24.3%) | χ^2^=0.017, df=1 | 0.896 |
| Any mental disorder^2^ | 104 (71.2%) | 235 (70.4%) | χ^2^=0.007, df=1 | 0.933 |
| Any trauma^3^ | 68 (64.8%) | 156 (70.6%) | χ^2^=0.869, df=1 | 0.351 |
| Self-reported psychopathology^4^ |  |  |  |  |
| Total (T-values) | 61.41 (9.99) | 60.27 (11.04) | t(283.03) = 1.12 | 0.262 |
| Internalizing (T-values) | 61.29 (10.27) | 60.44 (11.04) | t(289.68) = 0.83 | 0.407 |
| Externalizing (T-values) | 58.03 (10.64) | 57.11 (11.48) | t(288.83) = 0.87 | 0.387 |
| Psychopathic traits^5^ | 112.23 (22.64) | 112.91 (22.56) | t(309.38) = -0.31 | 0.752 |
| Non-verbal reasoning (IQ)^6^ | 95.49 (14.58) | 96.66 (14.22) | t(252.45) = -0.77 | 0.442 |
| *Notes.* IQ = intelligence quotient; total N of the tests performed vary between 326-592.  ^1^ assessed with the semi-structured clinical interview SCID-II.  ^2^ assessed with the semi-structured clinical interview Kiddie-SADS.  ^3^ assessed with the Essen Trauma-Inventory (ETI-KJ).  ^4^ Dimensional psychopathology was self-reported by participants at baseline with the Youth Self Report (YSR) and the Young Adult Self Report (YASR) of the Achenbach scales, t-values reported.  ^5^ Youth Psychopathic Traits Inventory (YPI) sum score.  ^6^ assessed with the Standard Progressive Matrices of Raven or the Culture Fair Intelligence Test. | | | | |

Table S2. Comparison of included (*N* = 185) versus non-included MAZ. participants (*N* = 412) regarding follow-up data of criminal records from the Swiss Federal Bureau of Statistics (BFS) up until the year 2017.

|  | *Included, N (%)* | *Non-included, N (%)* | *Test-statistic* | *p-value* |
| --- | --- | --- | --- | --- |
| **Before the age of 18** |  |  |  |  |
| *Any offense* | 81 (45.0%) | 176 (42.7%) | *χ^2^=0.181, df=1* | *0.671* |
| *Violent offense* | 27 (15.0%) | 65 (15.8%) | *χ^2^=0.014, df=1* | *0.907* |
| *Prison sentence* | 13 (7.2%) | 19 (4.6%) | *χ^2^=1.198, df=1* | *0.274* |
| **After the age of 18** |  |  |  |  |
| *Any offense* | 59 (32.8%) | 137 (33.3%) | *χ^2^=0.000, df=1* | *0.986* |
| *Violent offense* | 19 (10.6%) | 42 (10.2%) | *χ^2^=0.000, df=1* | *0.999* |
| *Prison sentence* | 28 (15.6%) | 44 (10.7%) | *χ^2^=2.350, df=1* | *0.125* |
| *Notes.* Any Offense covers delicts classified as “Vergehen” or “Verbrechen” in Swiss criminal law. We used the known data of the delict and not of the verdict for age classification. More information on the data from the Swiss federal office of Statistics are reported in Jäggi et al., 2021, [www.doi.org/10.1186/s13034-020-00355-1](http://www.doi.org/10.1186/s13034-020-00355-1) | | | | |

Table S3. Prevalence (in %) of different childhood maltreatment types across the years 3 to 18.

| ***Age***  ***Type*** | **3** | **4** | **5** | **6** | **7** | **8** | **9** | **10** | **11** | **12** | **13** | **14** | **15** | **16** | **17** | **18** |
| --- | --- | --- | --- | --- | --- | --- | --- | --- | --- | --- | --- | --- | --- | --- | --- | --- |
| **Parental neglect** | 89.73 | 89.19 | 89.73 | 89.73 | 90.27 | 90.27 | 90.81 | 91.89 | 91.89 | 92.43 | 92.43 | 91.89 | 91.89 | 91.89 | 91.35 | 90.81 |
| **Parental abuse** | 31.89 | 36.22 | 40.54 | 50.81 | 50.81 | 56.22 | 58.92 | 61.08 | 60.54 | 65.41 | 63.78 | 63.24 | 61.62 | 58.92 | 53.51 | 52.43 |
| **Sexual abuse** | 2.16 | 2.70 | 3.78 | 4.86 | 5.95 | 3.78 | 2.16 | 3.78 | 2.70 | 5.41 | 9.19 | 10.27 | 7.57 | 4.32 | 4.86 | 4.32 |
| **Peer violence** | 3.24 | 5.41 | 9.73 | 18.92 | 27.57 | 32.43 | 32.43 | 35.68 | 38.92 | 45.41 | 42.16 | 40.54 | 35.14 | 27.03 | 21.08 | 19.46 |

Figure S1. Correlation matrix of study variables.

**Table S4.** Variable importance of variables that predicted overall mental health problems. Significant predictors (*p* < .05) are marked in bold.

| *Predictor* | *Variable Importance* | *SD* | *p-value* |
| --- | --- | --- | --- |
| **MACE_SUM** | **2.7214706501** | **4.797648e-01** | **0.0008** |
| **MACE_MULTI** | **1.1785206265** | **1.620905e-01** | **0.0090** |
| MACE_Duration | 0.2112715411 | 3.210880e-02 | 0.2624 |
| MACE_NEGLECT_3 | 0.0100777743 | 2.258861e-02 | 0.5128 |
| MACE_NEGLECT_4 | 0.0191967465 | 2.266270e-02 | 0.4992 |
| MACE_NEGLECT_5 | -0.0845352893 | 1.217632e-02 | 0.6952 |
| MACE_NEGLECT_6 | -0.0668910229 | 1.960175e-02 | 0.6526 |
| MACE_NEGLECT_7 | -0.0347706657 | 2.004389e-02 | 0.6018 |
| MACE_NEGLECT_8 | -0.0784457053 | 1.545442e-02 | 0.6814 |
| MACE_NEGLECT_9 | -0.0762632949 | 3.081967e-02 | 0.6792 |
| MACE_NEGLECT_10 | -0.0741253689 | 1.581127e-02 | 0.6694 |
| MACE_NEGLECT_11 | -0.0505685922 | 1.487698e-02 | 0.6208 |
| MACE_NEGLECT_12 | -0.0852037271 | 1.024972e-02 | 0.6760 |
| MACE_NEGLECT_13 | 0.0050415562 | 2.028712e-02 | 0.5196 |
| MACE_NEGLECT_14 | -0.0078628643 | 1.440524e-02 | 0.5484 |
| MACE_NEGLECT_15 | -0.0327123040 | 1.547894e-02 | 0.5840 |
| MACE_NEGLECT_16 | -0.0821362090 | 1.849704e-02 | 0.6886 |
| MACE_NEGLECT_17 | -0.1181161309 | 1.317320e-02 | 0.7350 |
| MACE_NEGLECT_18 | 0.0568810573 | 3.120806e-02 | 0.4202 |
| **MACE_ABUSE_3** | **1.5103647368** | **1.794950e-01** | **0.0016** |
| MACE_ABUSE_4 | 0.5473400446 | 8.960421e-02 | 0.0516 |
| **MACE_ABUSE_5** | **0.7892118676** | **8.040425e-02** | **0.0230** |
| **MACE_ABUSE_6** | **1.6250880756** | **1.500503e-01** | **0.0016** |
| **MACE_ABUSE_7** | **0.7494622946** | **9.217969e-02** | **0.0250** |
| MACE_ABUSE_8 | 0.5532192998 | 7.680796e-02 | 0.0566 |
| MACE_ABUSE_9 | 0.4484280932 | 8.570807e-02 | 0.0788 |
| MACE_ABUSE_10 | 0.2089327600 | 5.471606e-02 | 0.2210 |
| MACE_ABUSE_11 | -0.1237900921 | 3.726703e-02 | 0.7136 |
| MACE_ABUSE_12 | -0.1075946908 | 2.540735e-02 | 0.6616 |
| MACE_ABUSE_13 | -0.0594891572 | 3.430498e-02 | 0.5918 |
| MACE_ABUSE_14 | 0.2120025296 | 3.316851e-02 | 0.2242 |
| MACE_ABUSE_15 | -0.0425155013 | 3.096178e-02 | 0.5574 |
| MACE_ABUSE_16 | -0.1085870699 | 3.859479e-02 | 0.6824 |
| MACE_ABUSE_17 | -0.0536016113 | 5.229847e-02 | 0.5750 |
| MACE_ABUSE_18 | 0.2260471942 | 5.379733e-02 | 0.1918 |
| MACE_SEXA_3 | 0.0000000000 | 0.000000e+00 | 1.0000 |
| MACE_SEXA_4 | 0.0000000000 | 0.000000e+00 | 1.0000 |
| MACE_SEXA_5 | 0.0003080754 | 2.869925e-05 | 0.0782 |
| MACE_SEXA_6 | 0.0016032788 | 1.594397e-04 | 0.2722 |
| MACE_SEXA_7 | 0.0498886617 | 3.375105e-03 | 0.1224 |
| MACE_SEXA_8 | 0.0004926912 | 8.059249e-05 | 0.0702 |
| MACE_SEXA_9 | 0.0000000000 | 0.000000e+00 | 1.0000 |
| MACE_SEXA_10 | 0.0000000000 | 0.000000e+00 | 0.9384 |
| MACE_SEXA_11 | 0.0000000000 | 0.000000e+00 | 1.0000 |
| MACE_SEXA_12 | 0.0000000000 | 0.000000e+00 | 0.6074 |
| MACE_SEXA_13 | -0.0195407226 | 1.726714e-03 | 0.5382 |
| MACE_SEXA_14 | 0.0226943031 | 4.127876e-03 | 0.3350 |
| MACE_SEXA_15 | -0.0058614197 | 1.223957e-03 | 0.4844 |
| MACE_SEXA_16 | 0.0014611298 | 6.449271e-05 | 0.1896 |
| MACE_SEXA_17 | 0.0296974878 | 2.226387e-03 | 0.1066 |
| **MACE_SEXA_18** | **0.0641362886** | **4.740012e-03** | **0.0202** |
| MACE_PEER_3 | 0.0000000000 | 0.000000e+00 | 1.0000 |
| MACE_PEER_4 | 0.0397262724 | 2.494511e-03 | 0.1282 |
| MACE_PEER_5 | 0.0951238313 | 7.595917e-03 | 0.1476 |
| MACE_PEER_6 | 0.1349449228 | 1.211834e-02 | 0.1816 |
| MACE_PEER_7 | -0.0262309098 | 5.332789e-03 | 0.5376 |
| MACE_PEER_8 | -0.0013548009 | 6.522754e-03 | 0.4950 |
| MACE_PEER_9 | 0.0029144404 | 1.064228e-02 | 0.4896 |
| MACE_PEER_10 | 0.0291251962 | 1.092029e-02 | 0.4206 |
| MACE_PEER_11 | 0.0128718044 | 1.645833e-02 | 0.4568 |
| MACE_PEER_12 | 0.0025204176 | 1.690131e-02 | 0.4676 |
| MACE_PEER_13 | 0.4750918242 | 5.250173e-02 | 0.0756 |
| MACE_PEER_14 | 1.2068946910 | 1.288806e-01 | 0.0090 |
| **MACE_PEER_15** | **0.7500732128** | **5.679457e-02** | **0.0200** |
| **MACE_PEER_16** | **0.7339189744** | **8.817598e-02** | **0.0204** |
| **MACE_PEER_17** | **0.7521548393** | **4.293085e-02** | **0.0154** |
| **MACE_PEER_18** | **1.1782415062** | **1.138179e-01** | **0.0060** |
| sex | -0.1218624121 | 2.244107e-02 | 0.6034 |
| age | 0.5156331076 | 8.509743e-02 | 0.0884 |

**Table S5.** Variable importance of variables that predicted internalizing mental health problems. Significant predictors (*p* < .05) are marked in bold.

| *Predictor* | *Variable Importance* | *SD* | *p-value* |
| --- | --- | --- | --- |
| **MACE_SUM** | **3.0180474786** | **4.039521e-01** | **0.0000** |
| **MACE_MULTI** | **2.0677835451** | **1.964934e-01** | **0.0004** |
| **MACE_Duration** | **0.9858738929** | **9.243744e-02** | **0.0362** |
| MACE_NEGLECT_3 | 0.1856515752 | 4.553614e-02 | 0.2586 |
| MACE_NEGLECT_4 | 0.4934450439 | 1.034780e-01 | 0.0730 |
| MACE_NEGLECT_5 | 0.1498614616 | 3.992202e-02 | 0.2806 |
| MACE_NEGLECT_6 | 0.2176930199 | 3.973717e-02 | 0.2094 |
| MACE_NEGLECT_7 | 0.1161009296 | 2.112314e-02 | 0.3392 |
| MACE_NEGLECT_8 | 0.1894238240 | 5.746552e-02 | 0.2388 |
| MACE_NEGLECT_9 | -0.0023169084 | 2.703152e-02 | 0.5308 |
| MACE_NEGLECT_10 | 0.2773905807 | 6.407135e-02 | 0.1660 |
| MACE_NEGLECT_11 | 0.0363268707 | 2.794134e-02 | 0.4612 |
| MACE_NEGLECT_12 | 0.0414395862 | 2.373977e-02 | 0.4524 |
| MACE_NEGLECT_13 | 0.1819313315 | 4.133075e-02 | 0.2550 |
| MACE_NEGLECT_14 | 0.1825332609 | 3.203278e-02 | 0.2482 |
| MACE_NEGLECT_15 | 0.3493741999 | 4.772696e-02 | 0.1234 |
| MACE_NEGLECT_16 | -0.0162047391 | 1.946082e-02 | 0.5692 |
| MACE_NEGLECT_17 | -0.0334353897 | 3.747630e-02 | 0.5830 |
| MACE_NEGLECT_18 | -0.0882141894 | 2.308289e-02 | 0.6602 |
| MACE_ABUSE_3 | 0.1360932793 | 2.524612e-02 | 0.2288 |
| MACE_ABUSE_4 | 0.0095654267 | 1.665769e-02 | 0.4548 |
| MACE_ABUSE_5 | 0.1671322669 | 4.865567e-02 | 0.2356 |
| **MACE_ABUSE_6** | **0.7828002498** | **9.080136e-02** | **0.0274** |
| MACE_ABUSE_7 | 0.3217193489 | 5.497477e-02 | 0.1334 |
| MACE_ABUSE_8 | 0.2505211112 | 3.514861e-02 | 0.1866 |
| MACE_ABUSE_9 | 0.0472063241 | 2.784335e-02 | 0.4260 |
| MACE_ABUSE_10 | 0.0341153655 | 5.614816e-02 | 0.4322 |
| MACE_ABUSE_11 | -0.1548644290 | 2.344437e-02 | 0.7618 |
| MACE_ABUSE_12 | -0.0689114081 | 1.550946e-02 | 0.5930 |
| MACE_ABUSE_13 | -0.0879260499 | 2.217160e-02 | 0.6392 |
| MACE_ABUSE_14 | 0.2033976526 | 5.064437e-02 | 0.2172 |
| MACE_ABUSE_15 | -0.0301026042 | 3.662381e-02 | 0.5250 |
| MACE_ABUSE_16 | 0.0858163729 | 3.418821e-02 | 0.3594 |
| MACE_ABUSE_17 | -0.0580019558 | 3.714699e-02 | 0.5834 |
| MACE_ABUSE_18 | 0.0858646860 | 5.283341e-02 | 0.3318 |
| MACE_SEXA_3 | 0.0000000000 | 0.000000e+00 | 1.0000 |
| MACE_SEXA_4 | 0.0000000000 | 0.000000e+00 | 1.0000 |
| MACE_SEXA_5 | 0.0006403110 | 1.504651e-05 | 0.0688 |
| MACE_SEXA_6 | 0.0085608762 | 6.629303e-04 | 0.2092 |
| MACE_SEXA_7 | 0.0106859434 | 7.795937e-04 | 0.2788 |
| MACE_SEXA_8 | 0.0006949634 | 2.414871e-05 | 0.0740 |
| MACE_SEXA_9 | 0.0000000000 | 0.000000e+00 | 1.0000 |
| MACE_SEXA_10 | -0.0004420833 | 9.771882e-06 | 0.9396 |
| MACE_SEXA_11 | 0.0000000000 | 0.000000e+00 | 1.0000 |
| MACE_SEXA_12 | -0.0002685082 | 2.028716e-04 | 0.6100 |
| MACE_SEXA_13 | -0.0025977015 | 1.166068e-03 | 0.4266 |
| MACE_SEXA_14 | -0.0004704172 | 4.518356e-03 | 0.4218 |
| MACE_SEXA_15 | -0.0090341603 | 2.024055e-03 | 0.5038 |
| MACE_SEXA_16 | 0.0000000000 | 0.000000e+00 | 0.8214 |
| **MACE_SEXA_17** | **0.0707679810** | **5.328623e-03** | **0.0410** |
| **MACE_SEXA_18** | **0.0895259932** | **1.035094e-02** | **0.0076** |
| MACE_PEER_3 | 0.0000000000 | 0.000000e+00 | 1.0000 |
| MACE_PEER_4 | 0.0001734084 | 8.567836e-05 | 0.3508 |
| MACE_PEER_5 | -0.0144143311 | 1.029040e-03 | 0.5422 |
| MACE_PEER_6 | -0.0408807717 | 3.040398e-03 | 0.5882 |
| MACE_PEER_7 | -0.0563182558 | 9.930241e-03 | 0.6020 |
| MACE_PEER_8 | -0.0333656584 | 9.511699e-03 | 0.5634 |
| MACE_PEER_9 | -0.0248960317 | 9.418862e-03 | 0.5288 |
| MACE_PEER_10 | 0.0194610498 | 1.181730e-02 | 0.4370 |
| MACE_PEER_11 | 0.0090046759 | 1.508084e-02 | 0.4560 |
| MACE_PEER_12 | -0.0083328174 | 2.353740e-02 | 0.4824 |
| MACE_PEER_13 | 0.5361702808 | 4.921102e-02 | 0.0604 |
| **MACE_PEER_14** | **1.1996501133** | **1.004573e-01** | **0.0070** |
| **MACE_PEER_15** | **0.6028456651** | **1.004758e-01** | **0.0364** |
| MACE_PEER_16 | 0.3233042196 | 3.879007e-02 | 0.0938 |
| MACE_PEER_17 | 0.3367811018 | 3.399244e-02 | 0.0672 |
| **MACE_PEER_18** | **0.5608229222** | **7.725368e-02** | **0.0318** |
| sex | 0.2567348493 | 4.653588e-02 | 0.1470 |
| **age** | **1.7834829052** | **1.769716e-01** | **0.0056** |

**Table S6.** Variable importance of variables that predicted externalizing mental health problems. Significant predictors (*p* < .05) are marked in bold.

| *Predictor* | *Variable Importance* | *SD* | *p-value* |
| --- | --- | --- | --- |
| **MACE_SUM** | **2.2159154474** | **3.068483e-01** | **0.0006** |
| **MACE_MULTI** | **0.8381726480** | **1.259900e-01** | **0.0246** |
| MACE_Duration | 0.0441872686 | 2.732733e-02 | 0.4574 |
| MACE_NEGLECT_3 | 0.0299713181 | 2.489206e-02 | 0.4778 |
| MACE_NEGLECT_4 | 0.0133481047 | 1.466065e-02 | 0.5144 |
| MACE_NEGLECT_5 | -0.0217819973 | 1.323584e-02 | 0.5712 |
| MACE_NEGLECT_6 | -0.0076946501 | 1.191086e-02 | 0.5508 |
| MACE_NEGLECT_7 | 0.0639343684 | 1.899777e-02 | 0.4194 |
| MACE_NEGLECT_8 | 0.0075943418 | 1.484649e-02 | 0.5214 |
| MACE_NEGLECT_9 | 0.0486431651 | 2.097652e-02 | 0.4720 |
| MACE_NEGLECT_10 | -0.0250434417 | 1.356704e-02 | 0.5824 |
| MACE_NEGLECT_11 | -0.0155844870 | 1.255731e-02 | 0.5748 |
| MACE_NEGLECT_12 | -0.0141844263 | 1.010239e-02 | 0.5536 |
| MACE_NEGLECT_13 | -0.0369466408 | 1.084265e-02 | 0.5940 |
| MACE_NEGLECT_14 | 0.0477636344 | 1.776841e-02 | 0.4418 |
| MACE_NEGLECT_15 | 0.0170695754 | 1.367063e-02 | 0.4956 |
| MACE_NEGLECT_16 | -0.0323163098 | 1.394354e-02 | 0.6024 |
| MACE_NEGLECT_17 | -0.0221516836 | 1.298468e-02 | 0.5666 |
| MACE_NEGLECT_18 | 0.0197594961 | 1.582394e-02 | 0.4860 |
| **MACE_ABUSE_3** | **3.5749108903** | **3.270546e-01** | **0.0000** |
| **MACE_ABUSE_4** | **1.4195452672** | **1.397188e-01** | **0.0032** |
| **MACE_ABUSE_5** | **1.2639571689** | **2.032901e-01** | **0.0044** |
| **MACE_ABUSE_6** | **1.2857107976** | **1.298287e-01** | **0.0050** |
| **MACE_ABUSE_7** | **0.8595901057** | **1.167183e-01** | **0.0212** |
| MACE_ABUSE_8 | 0.4172500556 | 7.294149e-02 | 0.0948 |
| MACE_ABUSE_9 | 0.3427126001 | 7.108776e-02 | 0.1240 |
| MACE_ABUSE_10 | 0.2042165512 | 3.445181e-02 | 0.2240 |
| MACE_ABUSE_11 | -0.0886270246 | 3.910327e-02 | 0.6594 |
| MACE_ABUSE_12 | 0.0084241228 | 3.311189e-02 | 0.4714 |
| MACE_ABUSE_13 | 0.3181298324 | 5.242568e-02 | 0.1440 |
| MACE_ABUSE_14 | 0.2079095031 | 5.157973e-02 | 0.2234 |
| MACE_ABUSE_15 | -0.0584072934 | 2.527443e-02 | 0.5808 |
| MACE_ABUSE_16 | -0.0443519616 | 2.925564e-02 | 0.5828 |
| MACE_ABUSE_17 | 0.2339351767 | 4.396896e-02 | 0.1934 |
| MACE_ABUSE_18 | 0.6212332477 | 7.231713e-02 | 0.0516 |
| MACE_SEXA_3 | 0.0000000000 | 0.000000e+00 | 1.0000 |
| MACE_SEXA_4 | 0.0000000000 | 0.000000e+00 | 1.0000 |
| MACE_SEXA_5 | 0.0009939131 | 6.088836e-05 | 0.0694 |
| MACE_SEXA_6 | 0.0003423149 | 5.813336e-06 | 0.2880 |
| MACE_SEXA_7 | -0.0011779160 | 5.715105e-04 | 0.5640 |
| MACE_SEXA_8 | 0.0002374310 | 2.818673e-06 | 0.0764 |
| MACE_SEXA_9 | 0.0000000000 | 0.000000e+00 | 1.0000 |
| MACE_SEXA_10 | 0.0000000000 | 0.000000e+00 | 0.9378 |
| MACE_SEXA_11 | 0.0000000000 | 0.000000e+00 | 1.0000 |
| MACE_SEXA_12 | -0.0010825945 | 2.874512e-05 | 0.6364 |
| MACE_SEXA_13 | -0.0172304488 | 1.665345e-03 | 0.5292 |
| MACE_SEXA_14 | -0.0140865244 | 1.692651e-03 | 0.5114 |
| MACE_SEXA_15 | -0.0226126881 | 1.069608e-03 | 0.6040 |
| MACE_SEXA_16 | 0.0040437693 | 2.206542e-04 | 0.1606 |
| MACE_SEXA_17 | 0.0381833790 | 2.759169e-03 | 0.0752 |
| **MACE_SEXA_18** | **0.0335681797** | **3.912721e-03** | **0.0428** |
| MACE_PEER_3 | 0.0000000000 | 0.000000e+00 | 1.0000 |
| MACE_PEER_4 | 0.0443072685 | 2.706931e-03 | 0.1052 |
| MACE_PEER_5 | 0.1718384411 | 1.213365e-02 | 0.0858 |
| MACE_PEER_6 | 0.1245555099 | 1.187672e-02 | 0.1954 |
| MACE_PEER_7 | -0.0395539392 | 6.385453e-03 | 0.5752 |
| MACE_PEER_8 | -0.0120921366 | 7.193451e-03 | 0.5040 |
| MACE_PEER_9 | -0.0519659863 | 9.671495e-03 | 0.6066 |
| MACE_PEER_10 | 0.0103037292 | 1.471188e-02 | 0.4612 |
| MACE_PEER_11 | -0.0010023292 | 1.192912e-02 | 0.4680 |
| MACE_PEER_12 | 0.0168544357 | 1.368730e-02 | 0.4256 |
| MACE_PEER_13 | 0.2630220416 | 4.037448e-02 | 0.1540 |
| **MACE_PEER_14** | **0.8526799089** | **8.105038e-02** | **0.0184** |
| **MACE_PEER_15** | **0.9024926786** | **8.875535e-02** | **0.0142** |
| **MACE_PEER_16** | **1.2046492997** | **1.075187e-01** | **0.0038** |
| **MACE_PEER_17** | **1.5489925908** | **1.321567e-01** | **0.0010** |
| **MACE_PEER_18** | **1.4293050191** | **2.188042e-01** | **0.0022** |
| sex | 0.0201478984 | 2.434042e-02 | 0.3622 |
| age | 0.6618763489 | 5.478150e-02 | 0.0610 |
